# Water decoction of *Pericarpium citri reticulatae* and *Amomi fructus* ameliorates alcohol-induced liver disease involved in the modulation of gut microbiota and TLR4/NF-κB pathway

**DOI:** 10.3389/fphar.2024.1392338

**Published:** 2024-06-20

**Authors:** Xing-Min Zhang, Yue-Chang Huang, Bai-Zhong Chen, Qian Li, Pan-Pan Wu, Wen-Hua Chen, Ri-Hui Wu, Chen Li

**Affiliations:** ^1^ School of Pharmacy and Food Engineering, Wuyi University, Jiangmen, China; ^2^ International Healthcare Innovation Institute (Jiangmen), Jiangmen, China; ^3^ Guangdong Provincial Key Laboratory of Large Animal Models for Biomedicine, Wuyi University, Jiangmen, China; ^4^ Guangdong Xinbaotang Biotechnology Co., Ltd., Jiangmen, China

**Keywords:** *Pericarpium citri reticulatae*, *Amomi fructus*, alcoholic liver disease, gut microbiota, oxidative stress, inflammatory response

## Abstract

**Introduction:**

Alcohol consumption alters the diversity and metabolic activities of gut microbiota, leading to intestinal barrier dysfunction and contributing to the development of alcoholic liver disease (ALD), which is the most prevalent cause of advanced liver diseases. In this study, we investigated the protective effects and action mechanism of an aqueous extraction of *Pericarpium citri reticulatae* and *Amomi fructus* (PFE) on alcoholic liver injury.

**Methods:**

C57BL/6 mice were used to establish the mouse model of alcoholic liver injury and orally administered 500 and 1,000 mg/kg/d of PFE for 2 weeks. Histopathology, immunohistochemistry, immunofluorescence, Western blotting, qRT-PCR, and 16S rDNA amplicon sequencing were used to analyze the mechanism of action of PFE in the treatment of alcohol-induced liver injury.

**Results:**

Treatment with PFE significantly improved alcohol-induced liver injury, as illustrated by the normalization of serum alanine aminotransferase, aspartate aminotransferase, total triglyceride, and cholesterol levels in ALD mice in a dose-dependent manner. Administration of PFE not only maintained the intestinal barrier integrity prominently by upregulating mucous production and tight junction protein expressions but also sensibly reversed the dysregulation of intestinal microecology in alcohol-treated mice. Furthermore, PFE treatment significantly reduced hepatic lipopolysaccharide (LPS) and attenuated oxidative stress as well as inflammation related to the TLR4/NF-κB signaling pathway. The PFE supplementation also significantly promoted the production of short-chain fatty acids (SCFAs) in the ALD mice.

**Conclusion:**

Administration of PFE effectively prevents alcohol-induced liver injury and may also regulate the LPS-involved gut–liver axis; this could provide valuable insights for the development of drugs to prevent and treat ALD.

## 1 Introduction

Alcohol use has been reported to cause approximately 3.3 million deaths annually, accounting for about 5.9% of all deaths globally (Thursz et al., 2018). Excessive consumption of alcohol induces alcoholic liver disease (ALD), which has become a major health concern in recent times, and its incidence has increased mainly among the youth (Tarantino et al., 2022). ALD can cause liver fibrosis, cirrhosis, and even liver cancer if appropriate interventions are not implemented (Zhou et al., 2020). Currently, the primary drugs used in the clinical treatment of liver diseases comprise corticosteroids, N-acetylcysteine (NAC), pentoxifylline, and silybin, etc (Ohashi et al., 2018). Among these, silybin is a natural compound known for its potent antioxidant activity and acts as one of the most effective agents in the clinical treatment of ALD with favorable tolerability. However, the limited solubility of silybin hampers its intestinal absorption efficiency and oral bioavailability, resulting in a series of side effects including gastrointestinal discomfort, headache, and pruritus (Bijak, 2017; Federico et al., 2017; Soleimani et al., 2019; Horowitz, 2022). Therefore, development of safer and more effective strategies to prevent and treat ALD holds critical importance.

The gut microbiota play central roles in the regulation of human physiology, metabolism, and nutrition. Alterations in the gut microbial community have been linked with the development of liver diseases induced by heavy drinking (Stärkel et al., 2018; Bajaj, 2019). Numerous studies have shown that alcohol intake can lead to intestinal dysbiosis and increase intestinal permeability (Konturek et al., 2018). Gut dysbiosis has been demonstrated to increase gut-derived endotoxins, such as lipopolysaccharide (LPS), and cause hepatic inflammation in alcohol-induced liver injuries (An et al., 2022). LPS can activate the toll-like receptor-4 (TLR4) and initiate a signaling cascade that results in the activation of the transcription factor nuclear factor-κB (NF-κB) and production of inflammatory cytokines (Wang et al., 2020a). The release of proinflammatory cytokines, such as tumor necrosis factor-α (TNF-α), interleukin-1β (IL-1β), and interleukin-6 (IL-6), can also be aggravated by excess reactive oxygen species (ROS) induced by alcohol exposure (O'Shannessy et al., 2015). Moreover, the consumption of alcohol disrupts fat metabolism by shifting the primary fuel source from fatty acids to ethanol, resulting in reduced oxidation of fatty acids and increased hepatic accumulation of fat (You and Arteel, 2019; Jeon and Carr, 2020). Controlling the origins of inflammation and regulating fat metabolism may therefore present viable strategies for ALD treatment.

Over the past few decades, numerous natural products and their derivatives have been shown to exhibit excellent biological activities against diabetes and liver diseases (Hu et al., 2024; Li et al., 2024). In particular, several natural products have been used in various traditional practices to prevent and treat ALD (Zhang et al., 2021a; Sun et al., 2023) owing to their minimal toxicity and comprehensive efficacy. Notably, different kinds of traditional Chinese medicines (TCMs), including the Jianpi Liqi Huoxue decoction, *Pueraria lobata*, and semen hoveniae extract, as well as some natural products like costunolide, *Coprinus comatus* polysaccharide, and Antarctic krill oil have been reviewed and demonstrated to improve alcohol-induced liver injury and steatosis. These effects have been shown to be closely associated with modulation of gut microbiota, suggesting that targeted modulation of gut microbiota by TCMs and natural products may be beneficial for ALD therapy (Zhu et al., 2023). *Pericarpium citri reticulatae* (PCR) and *Amomi fructus* (FA) are two commonly known medicinal and food homologous plants that are abundant in active ingredients, such as polyphenols, polysaccharides, and flavones. These main active ingredients have shown various excellent hepatoprotective properties in different environments. For instance, flavonoid-enriched PCR extracts have been found to inhibit liver injuries induced by different factors like cyclophoshamide (CTX) and CCl_4_ (Gao et al., 2023). Another recent study has systematically explored the mechanism of *Citri Reticulatae Pericarpium* as anti-liver injury approach, independently from the damage origin (Wu et al., 2021). Our previous study also revealed that polysaccharides from PCR could effectively improve high-fat-diet-induced liver steatosis by reshaping the gut microbiota in mice (Li et al., 2023). FA is widely used as an anti-inflammatory TCM in the treatment of gastrointestinal diseases owing to its capacity for heat clearing (Cai et al., 2021). Volatile oils from FA have been found to not only prevent 5-fluorouracil-induced intestinal mucositis by reducing the abundance of pathogenic bacteria and increasing the amount of probiotics (Zhang et al., 2017) but also inhibit non-alcoholic fatty liver disease (NAFLD) via the gut–liver axis (Lu et al., 2018). These studies have expanded the applications of PCR and FA in the study of TCMs. Importantly, PCR and FA act as a classical herb pair in TCM and have been used for thousands of years to clinically reinforce “qi,” invigorate the spleen, dry dampness, eliminate phlegm, and promote urination. There is increasing evidence supporting the fact that there may be various common mechanisms underlying both ALD and NAFLD (Tarantino and Citro, 2024). The hepatoprotective effects of PCR and FA on non-alcoholic liver injury have been demonstrated individually; however, the potentially synergistic effects of these two food-homologous plants against alcoholic liver injury remain unclear.

In the present study, *in vitro* enzyme experimental models were established to optimize the combination of PCR and FA (PFE). The synergistic effects of PCR and FA against alcoholic liver injury were then investigated in alcohol-treated mice. Classical molecular biology techniques were used to reveal the hepatoprotective potential mechanisms of PFE. These findings are expected to provide novel insights into the prevention and treatment of ALD.

## 2 Materials and methods

### 2.1 PFE extraction

PCR (collected in the year 2019) was provided by Guangdong Xinbaotang Biotechnology Co., Ltd. (Jiangmen, China). FA was obtained from Guangdong Huiqun Traditional Chinese Medicine Slices Co., Ltd. (Shantou, China). The PCR and FA samples were identified by Prof. Li-She Gan of Wuyi University for authenticity. Extractions of different ratios of PCR and FA were prepared (1:1, 1:2, and 2:1 by weight) in a 1:20 ratio of material to liquid. The mixture was soaked in water for 1 h and decocted for another 1 h. The obtained filtrates were diluted with 10 times the volume of water and decocted for another 30 min before filtering to obtain another filtrate. These diluted filtrates were combined and dehydrated using a rotary evaporator (R-210, Buchi Labortechnik AG, Switzerland), followed by drying in a lyophilizer (Jiangsu Saifei Medical Device Co., Ltd., Jiangsu, China).

### 2.2 Determination of *in vitro* enzymatic and antioxidant activities

Alpha-diphenyl-β-picrylhydrazyl (DPPH) and 2,2-azino-bis (3-ethylbenzothiazoline-6-sulfonic acid) (ABTS) were used to evaluate the antioxidant capacity of PFE according to the methods reported in literature with slight modifications (Liu et al., 2022a). Vitamin C (VC) was used as the positive control. The activities of alcohol dehydrogenase (ADH) and acetaldehyde dehydrogenase (ALDH) were determined using commercially available kits according to the manufacturer’s instructions, for which HaiWangJinZunPian (HWJZP) was used as the positive control.

### 2.3 Experimental design and drug administration

Seven-week-old male C57BL/6 mice (18–20 g bodyweight) were purchased from Zhuhai BesTest Bio-Tech Co., Ltd. (Zhuhai, China). The mice were housed in a specific-pathogen-free (SPF) facility under controlled conditions at a temperature of 25°C ± 2°C, light/dark cycle of 12/12 h, and relative humidity of 50% ± 10%. The mice were allowed free access to water and a standard diet *ad libitum*. After 1 week of adaptation, all animals were randomly assigned into five groups as follows: normal control group (NC), model group (Alcohol), silybin group (Sly), low-dose group (PFE-L, 500 mg/kg/d), and high-dose group (PFE-H, 1,000 mg/kg/d). Intragastric administration was performed once daily with sterile water or silybin at a dosage of 100 mg/kg or PFE at dosages of 500 mg/kg or 1,000 mg/kg for 14 days. For the alcohol exposure, from day 8, the mice were orally administered 20% alcohol (3 days), 40% alcohol (2 days), and 50% alcohol (2 days). Twelve hours after the last alcohol administration, the mice were humanely euthanized with CO_2_, and the serum, liver, and ileum were collected for further analyses. All animal-related experiments were ethically approved by the Committee on the Ethics of Animal Experiments of the International Healthcare Innovation Institute (Jiangmen, China) and consistently conducted in accordance with the institutional Guidelines for the Care and Use of Laboratory Animals.

### 2.4 Biochemical analysis

The blood samples were centrifuged at 3,500 rpm for 10 min at 4°C to collect the sera. The liver tissues were homogenated with saline in a ratio of 1:9 and centrifuged at 3,500 rpm for 10 min to obtain the supernatant. The levels of alanine aminotransferase (ALT) and aspartate aminotransferase (AST) (Mindray Bio-Medical Electronics Co., Ltd., Shenzhen, China) were determined as per previous methods with slight adjustments (Wang et al., 2021a). The total triglyceride (TG), total cholesterol (TC), high-density lipoprotein cholesterol (HDL-C), and low-density lipoprotein cholesterol (LDL-C) levels (Mindray Bio-Medical Electronics Co., Ltd., Shenzhen, China) in the serum or liver were tested as per previously described methods (Shuai et al., 2021; Zhang et al., 2023b). The malondialdehyde (MDA), glutathione (GSH), superoxide dismutase (SOD), and glutathione peroxidase (GSH-Px) levels were determined using commercially available kits (Nanjing Jiancheng Bioengineering Institute, Nanjing, China) according to reported methods with slight modifications (Xing et al., 2021; Liu et al., 2023). The LPS, TNF-α, IL-6, and IL-1β levels in the liver samples were analyzed with ELISA kits (Ruida Henghui Technology Development Co., Ltd., Beijing, China) as per previously reported methods with slight modifications (Yang et al., 2020; Li et al., 2021a).

### 2.5 Histopathological analysis

The histopathological examinations were performed according to previous reports (Jiang et al., 2020; Tao et al., 2022). In brief, the liver and ileum tissues were fixed in 4% paraformaldehyde for 24 h and prepared for paraffin sectioning and cryosectioning. Then, the 5-μm paraffin sections were deparaffinized with xylene and rehydrated with gradient alcohol, and 10-μm-thick cryosections were prepared by embedding in Tissue-Tek O.C.T. compound. The paraffin sections and cryosections of the liver and ileum were stained with hematoxylin and eosin (H&E), oil red O, or alcian blue (Servicebio, Wuhan, China), and images were captured with an orthographic biomicroscope (Olympus Corporation, Tokyo, Japan).

### 2.6 Quantitative real-time PCR (qRT-PCR)

The mRNA expressions of the genes were analyzed by qRT-PCR as reported before (Ding et al., 2021; Liu et al., 2022b; Hao et al., 2022). Briefly, the total RNA was extracted from the liver and ileum tissues using TRIzol^®^ Reagent (Life Technology, CA, United States). Then, cDNA synthesis was performed using a cDNA reverse transcription kit (Kailian Biotechnology Co., Ltd., Shengzhen, China) following the manufacturer’s instructions. Quantitative real-time PCR (qRT-PCR) analysis was performed using the 2× SYBR Green qPCR Master Mix (Saiweier Biotechnology Co., Ltd., Wuhan, China) with the target primers. The primer sequences of the relevant genes are listed in Table 1. The relative expressions of the target genes were normalized with that of β-actin.

**TABLE 1:** Primer sequences of the target genes used for qRT-PCR.

Primers	Forward primer	Reverse primer
TNF-α	TAG​CCA​GGA​GGG​AGA​ACA​GA	CCA​GTG​AGT​GAA​AGG​GAC​AGA
IL-1β	GCC​CAT​CCT​CTG​TGA​CTC​AT	AGG​CCA​CAG​GTA​TTT​TGT​CG
IL-6	TTC​TTG​GGA​CTG​ATG​CTG​GTG	CAC​AAC​TCT​TTT​CTC​ATT​TCC​ACG​A
IL-10	AAG​AAG​GCA​TGC​ACA​GCT​CA	AAG​TGG​GTG​CAG​CTG​TTC​TC
IL-18	ACT​GTA​GAG​ATA​ATG​CAC​CCC​G	AGT​TAC​AGC​CAT​ACC​TCT​AGG​C
Caspase-1	AGA​CAT​CCC​ACA​ATG​GGC​TC	TGA​AAA​TCG​AAC​CTT​GCG​GAA​A
NLRP3	CTC​CAA​CCA​TTC​TCT​GAC​CAG	ACA​GAT​TGA​AGT​AAG​GCC​GG
iNOS	CGC​TTG​GGT​CTT​GTT​CAC​T	TCT​TTC​AGG​TCA​CTT​TGG​TA
Arginase-1	GCT​TGC​TTC​GGA​ACT​CAA​C	CGC​ATT​CAC​AGT​CAC​TTA​GG
Occludin	GCG​GAA​GAG​GTT​GAC​AGT​CC	ACT​CCC​CAC​CTG​TCG​TGT​AG
ZO-1	CAGCCGCATCTTCTTGTG	AGG​AGC​GAG​ACC​CCA​CTA​A
β-Actin	GGC​TGT​ATT​CCC​TCC​ATC​G	CCA​GTT​GGT​AAC​AAT​GCC​ATG

### 2.7 Immunofluorescence (IF) staining

Immunofluorescence staining was performed to detect the expressions of ileal ZO-1 and occludin following previously described methods with slight modifications (Zhang et al., 2020; Zhang et al., 2021b; Zhou et al., 2021). In brief, after deparaffinization of the paraffin sections, antigen retrieval was implemented using ethylenediamine tetraacetic acid (EDTA) buffer (10 mM Tris, 1 mM EDTA, pH 9.0) in a microwave. The antigen-repaired sections were then incubated with primary antibodies ZO-1 and occludin (1:2000; Servicebio, Wuhan, China) overnight at 4°C. After thorough washing steps, secondary horseradish peroxidase (HRP)-conjugated anti-rabbit antibody (Servicebio, Wuhan, China) was incubated at room temperature for 50 min under dark conditions. Following three washes with tris-buffered saline with 0.1% Tween (TBST), the samples were restained with DAPI (Servicebio, Wuhan, China) for 10 min. The slides were viewed under an orthotopic biomicroscope and quantified using ImageJ software (National Institutes of Health, Bethesda, MD, United States).

### 2.8 Immunohistochemical (IHC) staining

Immunohistochemical (IHC) staining of the tissues was performed as per previous studies (Pei et al., 2021; Chen et al., 2022). Briefly, the deparaffinized sections were incubated in 3% hydrogen peroxide solution at room temperature for 25 min. Subsequently, the sections were washed three times with phosphate-buffered saline (PBS) for 5 min each time and blocked with 3% bovine serum albumin and 0.5% Triton X-100 in PBS. Then, the primary antibodies against TNF-α, IL-1β, and IL-6 (Servicebio, Wuhan, China) were incubated overnight at 4°C. Thereafter, secondary HRP-conjugated anti-rabbit antibody (1:300, Servicebio, Wuhan, China) was incubated for 50 min at room temperature followed by counterstaining with DAB (Servicebio, Wuhan, China) and hematoxylin. The sections were observed with an orthographic biomicroscope and quantified using ImageJ software (National Institutes of Health, Bethesda, MD, United States).

### 2.9 Western blot analysis

Western blot analysis was performed to determine the expressions of the proteins, as described in previous reports (Wang et al., 2021a; Zhang et al., 2021c; Ding et al., 2023). In brief, 200 mg of the liver tissue was lysed with 1 mL RIPA buffer (Beyotime, Shanghai, China) and centrifuged at 10,000 r/min and 4°C for 10 min. The supernatant was then collected, and the protein concentration was determined using the BCA kit (Aidlab, Beijing, China). Each sample was next mixed with a loading buffer in the volume ratio of 4:1 and denatured at 100°C for 5 min. Subsequently, the samples were resolved on 12% or 10% SDS-PAGE gels before being transferred onto nitrocellulose membranes. The membranes were then blocked using 5% skim milk in TBST at room temperature for 2 h. The primary antibodies TLR4, IκB, and NF-κB (1:1,000, Abcam, Cambridge, MA, United States) were incubated overnight at 4°C, followed by incubation of the secondary HRP-conjugated anti-rabbit antibody (1:1,000, Abcam, Cambridge, MA, United States) for 2 h. The GeIView 1,500 system was used to image the membranes, and ImageJ software was utilized to quantitatively analyze the protein bands with β-actin protein as the internal standard.

### 2.10 Gut microbiota analysis

The total fecal DNA was extracted using the QIAamp-DNA Stool Mini Kit (Qiagen, Hilden, Germany). The integrity, quantity, and quality of the extracted DNA were examined by electrophoresis in 1% (wt/vol) agarose gels with an Agilent Bioanalyzer 2100 system (Agilent Technologies, San Diego, CA, United States). The 16S rRNA gene amplicon was sequenced as described previously (Li et al., 2021b; Wang et al., 2023). The V3–V4 regions of the bacterial 16S rRNA genes were amplified using the 338F/806R primers on the ABI GeneAmp^®^9700 PCR System (Applied Biosystems, Foster City, CA, United States). The PCR amplification products were then purified using Agencourt AMPure XP magnetic beads and eluted in an elution buffer before being quantified by a QuantiFluorTM-ST Handheld Fluorometer with UV/blue channels (Promega Corporation, Madison, WI, United States). A Nextera kit set A (Illumina, San Diego, CA, United States) was used to prepare the DNA library, and the qualified libraries were selected for sequencing on an Illumina HiSeq platform at HuaDa Gene Technology Co., Ltd. (Shenzhen, China) in accordance with the manufacturer’s instructions. The filtered and trimmed sequencing data were classified into operational taxonomic units (OTUs) based on sequence similarity (>97%) by using UCLUST software. The generated valid sequences were assigned taxonomy using the SILVA database (Release115 http://www.arb-silva.de). The Quantitative Insights Into Microbial Ecology (QIIME) software package was then used to analyze the sequencing results in line with a previous study, with slight modifications (Li et al., 2023). The MOTHUR program was next used to analyze the rarefaction curves and calculate the richness estimators and diversity indices. The linear discriminant analysis effect size (LEfSe) was performed to identify the significantly difference species.

### 2.11 Determination of short-chain fatty acids (SCFAs) by gas chromatography mass spectrometry (GC-MS)

The metabonomics was performed according to a previous method with slight modifications (Jiang et al., 2021). In brief, about 200 mg/mL of the cecal content was homogenized, vortexed, mixed, and incubated on ice for 30 min. After centrifugation at 10000 *g*/min for 10 min, 0.5 mL of the supernatant was removed and mixed with 0.1 mL of metaphosphoric acid solution (25%) and 11.65 μL crotonic acid solution (210 mmol/L) at 4°C for 30 min. The mixture was then centrifuged at 8,000 *g*/min for 10 min to obtain the purified filtrate and injected into a GC-MS apparatus for analysis. The chromatographic conditions included a temperature of 260°C, volume of 1 μL, and rate of 0.5 mL/min. The time-setting program started with an initial temperature of 100°C maintained for 5 min, followed by increasing to 190°C at 20°C/min and maintaining for l min, and finally increased to 260°C at 20°C/min. The entire program required about 13.5 min for execution.

### 2.12 Statistical analysis

The quantitative data were expressed as means ± standard deviations (SDs). The significance between multiple groups with only one dependent variable was analyzed using one-way analysis of variance (ANOVA) followed by Dunnett’s multiple-comparison test in SPSS 26.0 software (IBM, New York, NY, United States). The results were visualized with Origin 2022 software (OriginLab, Massachusetts, United States). The significance level was set at *p* < 0.05.

## 3 Results

### 3.1 *In vitro* enzymatic activity and antioxidant assays of PFE

**FIGURE 1:**
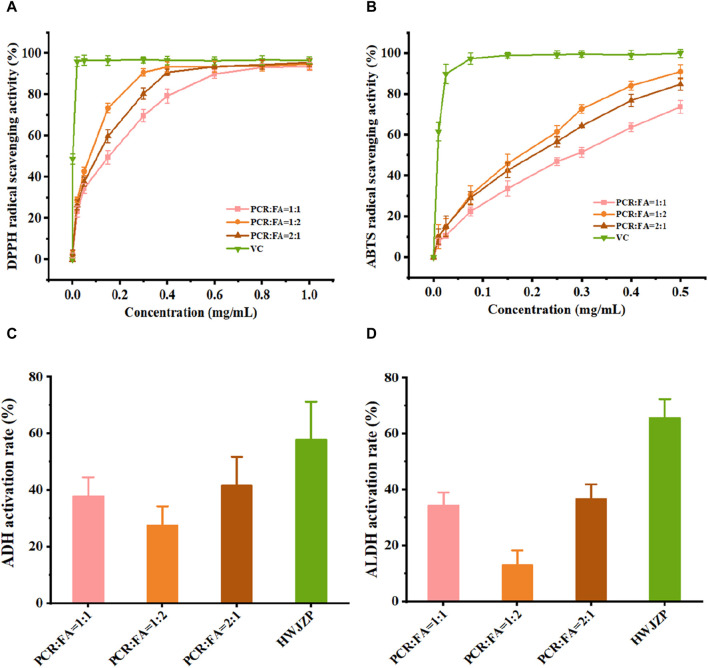
PFE differentially enhances the *in vitro* activities of enzymes and antioxidants. **(A)** DPPH radical scavenging activity. **(B)** ABTS radical scavenging activity. **(C)** Alcohol dehydrogenase (ADH) activity. **(D)** Acetaldehyde dehydrogenase (ALDH) activity. ABTS: 2,2-azino-bis (3-ethylbenzothiazoline-6-sulfonic acid), DPPH: alpha-diphenyl-β-picrylhydrazyl, PCR: *Pericarpium citri reticulatae*, FA: *Amomi fructus*, VC: vitamin C, HWJZP: HaiWangJinZunPian.

As the ratio of PCR to FA in the PFE might affect the pharmacological evaluations, we first screened for the optimal ratio of PCR to FA. The extractions of PCR and FA (PFE) were prepared with ratios of 1:1, 1:2, and 2:1 by weight. The DPPH and ABTS radical scavenging activities of PCR and FA were sequentially enhanced in the ratio order of 1:1, 2:1, and 1:2 (Figures 1A, B), indicating that PFE has strong antioxidant activity. However, the activities of ethanol dehydrogenase and acetaldehyde dehydrogenase *in vitro* for PCR and FA were sequentially enhanced in the ratio order of 1:2, 1:1, and 2:1 (Figures 1C, D), suggesting that PFE could promote alcohol metabolism. By taking into account the results of both antioxidant and enzymatic activities, PFE in the ratio of 2:1 was selected for *in vivo* evaluations in the subsequent experiments.

### 3.2 PFE improves lipid metabolism disorders in alcohol-fed mice

**FIGURE 2:**
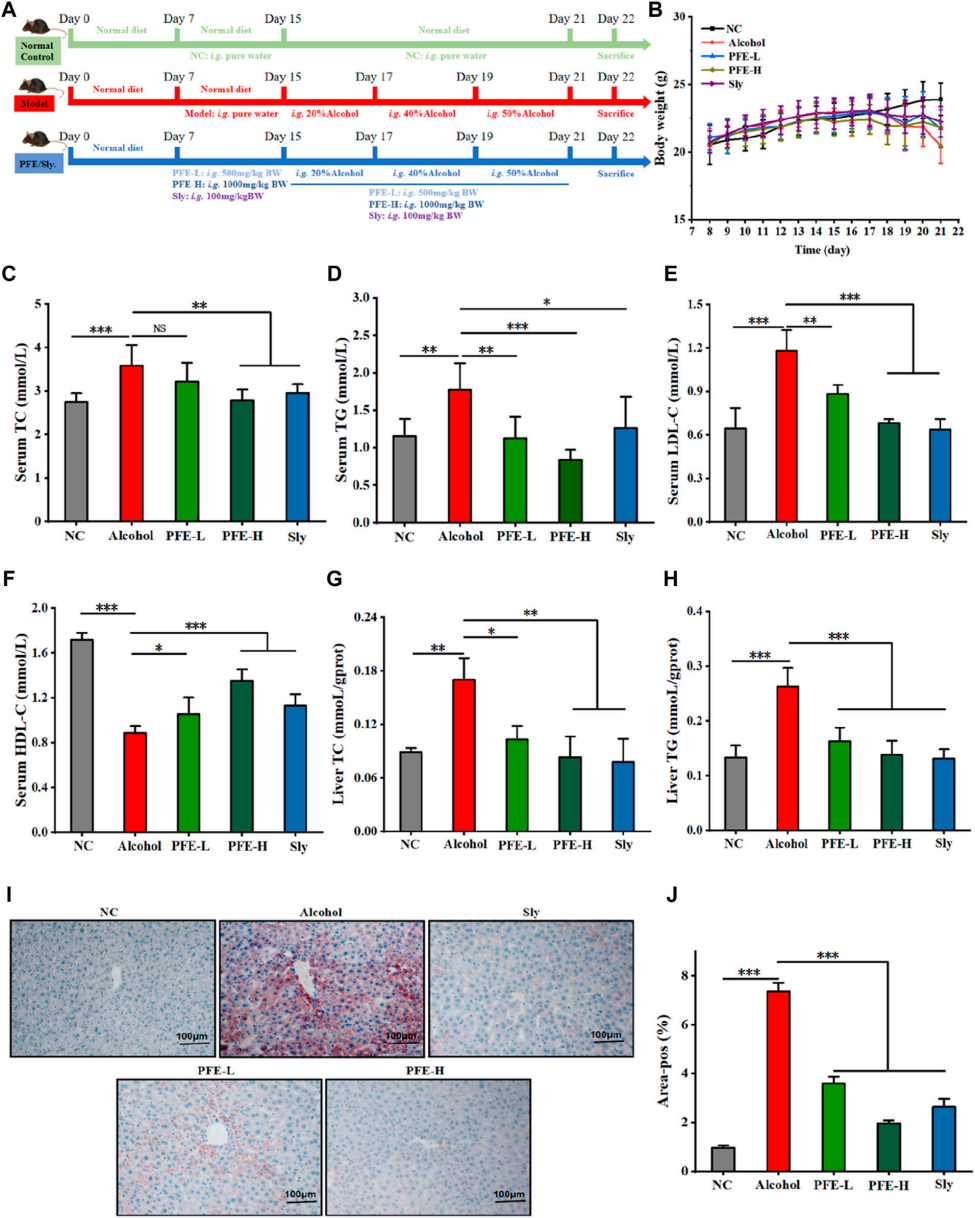
Treatment with PFE improves lipid metabolism disorders induced by alcohol feeding in mice. **(A)** Schematic representation of the intervention schedules for the different experimental groups. **(B)** Bodyweights of the mice. **(C,D)** Serum concentrations of total cholesterol (TC) and total triglyceride (TG). **(E,F)** Serum levels of low-density lipoprotein cholesterol (LDL-C) and high-density lipoprotein cholesterol (HDL-C). **(G,H)** Liver TC and TG levels. **(I,J)** Representative images of oil red O staining for liver tissues and their quantification. The data are expressed as means ± SD (*n* = 6–8). Statistical significance was determined by comparison with the alcohol group; ^*^ *p* < 0.05, ^**^ *p* < 0.01, and ^***^ *p* < 0.001. NS denotes non-significant. NC: normal control group, Alcohol: model group, Sly: silybin group, PFE-L: low-dose group, PFE-H: high-dose group.

To test whether PFE could improve liver injuries induced by excess alcohol intake, the experimental mice were orally treated with or without alcohol in combination with or without PFE for 3 weeks (Figure 2A). Compared with the controls, the mice subjected to alcohol feeding exhibited downward trends in their bodyweights (Figure 2B). The bodyweights of mice exposed to alcohol were maintained to a certain extent when compared to those not exposed to alcohol. Compared to NC mice, the alcohol-fed mice showed significantly increased levels of TG, TC, and LDL-C, as well as a clearly decreased level of HDL-C in serum (Figures 2C–F). Accordingly, the PFE treatment not only effectively reduces serum levels of TG, TC, and LDL-C but also elevates the serum HDL-C level in mice exposed to alcohol in a dose-dependent manner (Figures 2C–F). The TC and TG levels in the mice liver tissues were determined to be consistent with the changes obtained from serum measurements (Figures 2G, H). Furthermore, an increase in the oil red O positive area of the liver was observed in the alcohol-fed mice, indicating that alcohol exposure induced lipid accumulation in the liver in mice. The alcohol-exposure-induced hepatic lipid accumulation was significantly reduced after treatment with PFE in the mice (Figures 2I, J). These results suggest that PFE can effectively regulate lipid metabolism disorders induced by alcohol, which could help ameliorate alcoholic fatty liver disease.

### 3.3 PFE alleviates liver injury and oxidative stress in alcohol-fed mice

**FIGURE 3:**
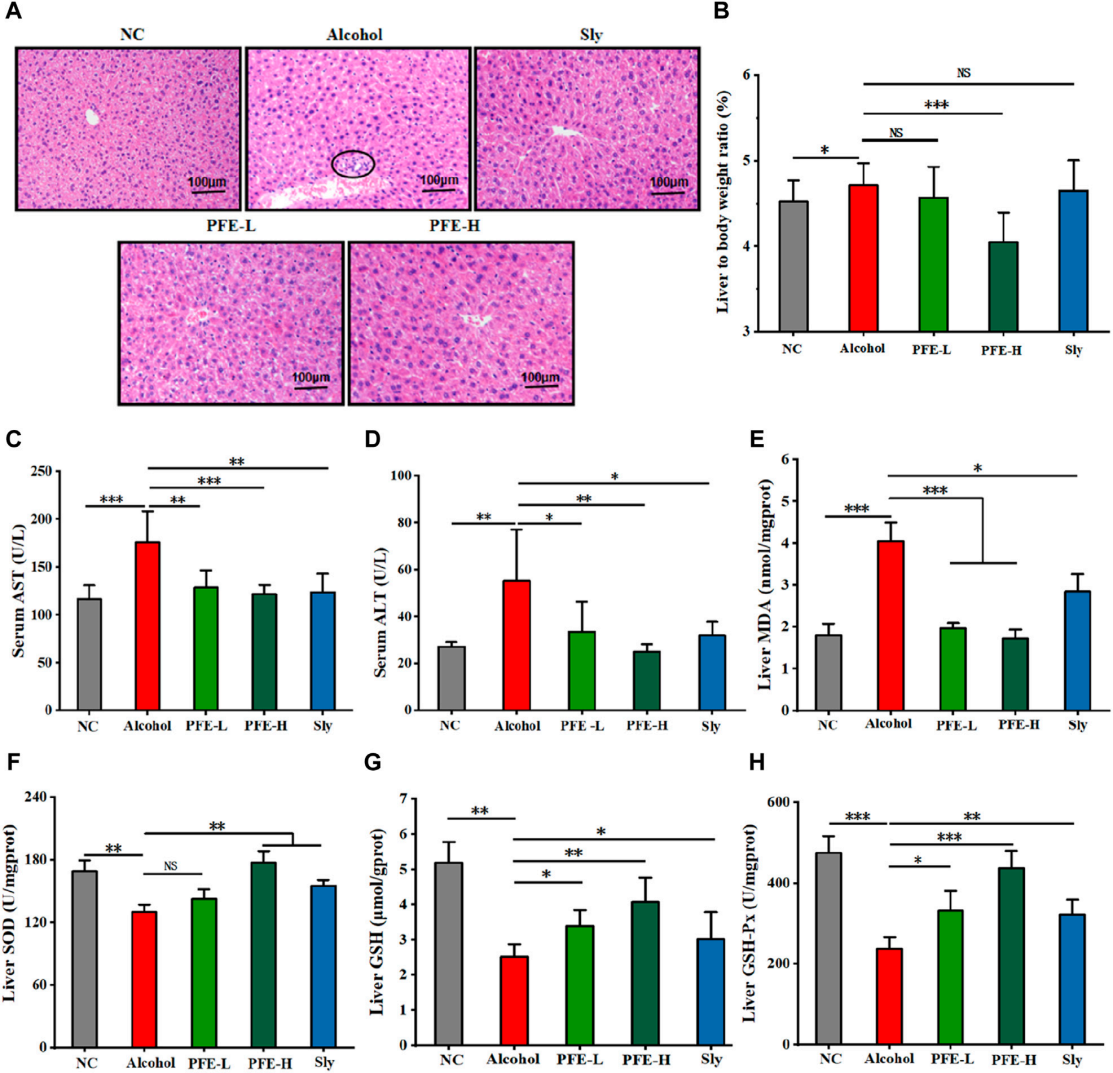
Supplementation with PFE mitigates liver injury and oxidative stress induced by alcohol exposure in mice. **(A)** Representative images of H&E staining for liver tissues. **(B)** Liver weight/bodyweight ratio. **(C,D)** Levels of serum aspartate aminotransferase (AST) and alanine aminotransferase (ALT). **(E)** Liver content of malondialdehyde (MDA). **(F–H)** Liver contents of superoxide dismutase (SOD), glutathione (GSH), and glutathione peroxidase (GSH-Px). The data are expressed as means ± SD. (*n* = 6–8). Statistical significance was determined by comparison with the alcohol group; ^*^ *p* < 0.05, ^**^ *p* < 0.01, and ^***^ *p* < 0.001. NS denotes non-significant. NC: normal control group, Alcohol: model group, Sly: silybin group, PFE-L: low-dose group, PFE-H: high-dose group.

To investigate the protective effects of PFE against alcohol-induced liver injury, we conducted histopathological examination of the liver tissues. The hepatocytes in the liver of the control mice exhibited well-preserved structural integrity around the central vein in a highly organized manner, with homogeneous cytoplasmic staining and clear nuclei (Figure 3A). In contrast, the hepatocytes in the liver of the alcohol-fed mice displayed enlarged sizes, cellular swelling, blurred cell boundaries, formation of aggregates (indicated by circles), and inflammatory cell infiltration (Figure 3A). Notably, the PFE treatment significantly revised these pathological changes induced by alcohol exposure, resulting in clearer demarcation between cells in the liver (Figure 3A). Furthermore, the ratio of liver weight to bodyweight was lower in the PFE-treated mice exposed to alcohol than in mice fed with alcohol alone (Figure 3B). Compared to the control mice, the key markers of liver injury, including serum AST and ALT, in the alcohol-fed mice were significantly increased by 1.5-fold and 2.0-fold, respectively (Figures 3C, D). Importantly, treatment with PFE significantly decreased the activities of serum AST and ALT in the alcohol-fed mice. Furthermore, mice exposed to alcohol showed significantly decreased activities of the antioxidant enzymes, including SOD, GSH, and GSH-Px in the liver, which were significantly reversed after PFE treatment in the livers of the alcohol-fed mice (Figures 3F–H). Meanwhile, MDA, a marker of lipid peroxidation, was significantly increased in the liver tissues of alcohol-fed mice relative to the control mice, which was also effectively reversed by PFE intervention (Figure 3E), indicating that PFE could enhance of the liver in alcohol-fed mice. The above results suggest that PFE alleviates alcohol-induced liver injury, which could be related to the strong antioxidant capacity against liver oxidative stress in mice.

### 3.4 PFE reduces LPS and inhibits TLR4/NF-κb-related inflammatory response

**FIGURE 4:**
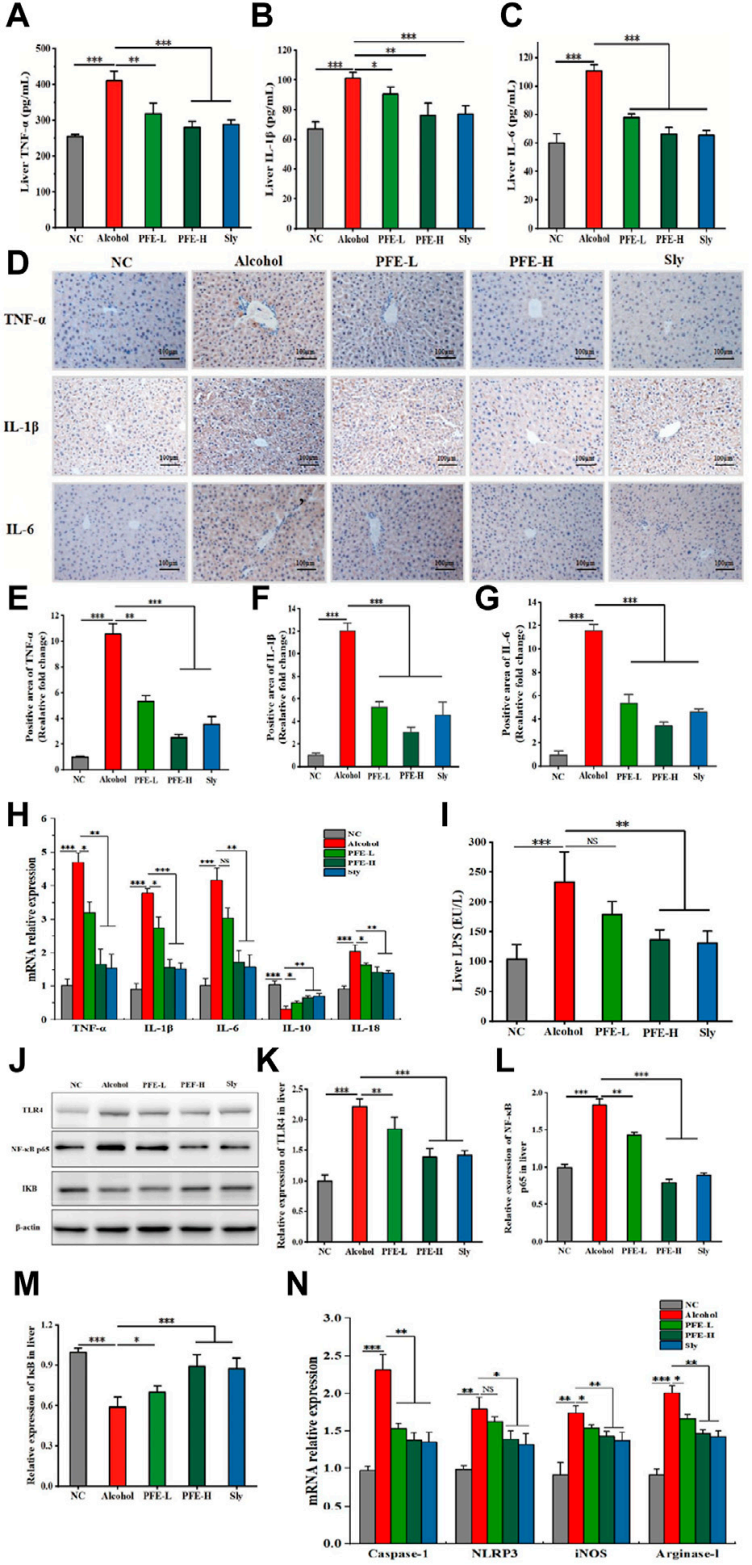
Administration of PFE inhibits inflammatory responses and the TLR4/NF-κB signaling pathway in mice with alcohol-induced liver injury. **(A–C)** Levels of tumor necrosis factor-α (TNF-α), interleukin-1β (IL-1β), and interleukin-6 (IL-6) in mice liver tissues. **(D)** Representative immunohistochemical images of inflammatory cytokines TNF-α, IL-1β, and IL-6 in mice liver tissues. **(E–G)** Quantitative analysis of the immunohistochemical staining for TNF-α, IL-1β, and IL-6 (*n* = 3). **(H)** mRNA relative expressions of TNF-α, IL-1β, IL-6, interleukin-10 (IL-10), and interleukin-18 (IL-18) in mouse liver determined by qRT-PCR. **(I)** Content of hepatic lipopolysaccharides (LPS) in mice. **(J–M)** Representative images of Western blot analysis for toll-like receptor 4 (TLR4), nuclear factor-κBp65 (NF-κBp65), and inhibitor of NF-κB (IκB) protein expressions in mouse liver and its relative quantitative analysis. **(N)** mRNA relative expressions of caspase-1, NOD-like receptor thermal protein domain associated protein 3 (NLRP3), inducible nitric oxide synthase (iNOS), and arginase-1 in mouse liver determined by qRT-PCR. The data are expressed as means ± SD (*n* = 6–8). Statistical significance was determined by comparison with the alcohol group; ^*^ *p* < 0.05, ^**^ *p* < 0.01, and ^***^ *p* < 0.001. NS denotes non-significant. NC: normal control group, Alcohol: model group, Sly: silybin group, PFE-L: low-dose group, PFE-H: high-dose group.

As severe injuries always evoke systemic inflammatory response, we next tested whether PFE showed anti-inflammatory activity against alcoholic liver injury by measuring the inflammatory cytokines. Compared to the controls, the TNF-α, IL-1β, and IL-6 levels were found to be elevated by approximately 1.6-fold, 1.5-fold, and 1.8-fold, respectively, in the livers of the alcohol-fed mice (Figures 4A–C). Interestingly, administration of PFE effectively reduced the hepatic contents of TNF-α, IL-1β, and IL-6 in the alcohol-induced mice in a dose-dependent manner (Figures 4A–C). These changes caused by PFE treatment in the alcohol-exposed mice were further confirmed by IHC results (Figures 4D–G). In line with the changes to the liver proteins, the mRNA expressions of TNF-α, IL-1β, and IL-6 were also significantly decreased in the alcohol-exposed mice treated with PFE compared to mice exposed to alcohol alone (Figure 4H). We also tested the hepatic LPS level, a well-characterized pathogen-associated molecular pattern that induces an inflammatory response via stimulation of the TLRs. Notably, the alcohol-fed mice showed high concentrations of LPS in their livers, but this was effectively reduced after PFE administration (Figure 4I).

As the synthesis and release of proinflammatory cytokines and chemokines are induced by activation of the TLR4/NF-κB signaling pathway, we tested the mechanistic path by which PFE modulates the gut–liver axis. Compared to the controls, alcohol exposure significantly upregulated the expressions of TLR4 and NF-κB p65 proteins as well as mRNA expressions of the NOD-like receptor thermal protein domain associated protein 3 (NLRP3) and caspase-1 while downregulating IκB protein expression in the liver (Figures 4J–N). Conversely, treatment with PFE-H resulted in substantial reductions in the hepatic TLR4, NF-κB, NLRP3, and caspase-1 expressions along with increased IκB protein expression in the alcohol-fed mice (Figures 4J–N), indicating that PFE amelioration of inflammation in ALD could be related to the suppression of the LPS-TLR4-NF-κB/NLRP3 axis. Because the microbial component of LPS can drive macrophage polarization toward the M1 phenotype macrophages that are mainly involved in proinflammatory responses (Chen et al., 2020), we wanted to determine whether PFE affected macrophage polarization. Compared with the controls, the mRNA expressions of IL-10 in the alcohol-exposed mice were significantly decreased, while the mRNA expressions of IL-18, inducible nitric oxide synthase (iNOS), and arginase-1 were significantly increased (Figures 4H, N). Interestingly, PFE administration dramatically increased hepatic mRNA expression of IL-10 and decreased hepatic mRNA expressions of IL-18, iNOS, and arginase-1 in the alcohol-fed mice (Figures 4H, N), indicating that PFE could inhibit macrophage polarization. Taken together, these findings suggest that PFE mitigates alcohol-induced liver inflammation by regulating macrophage polarization and suppressing the LPS-TLR4-NF-κB/NLRP3 axis.

### 3.5 PFE repairs intestinal barrier in alcohol-fed mice

**FIGURE 5:**
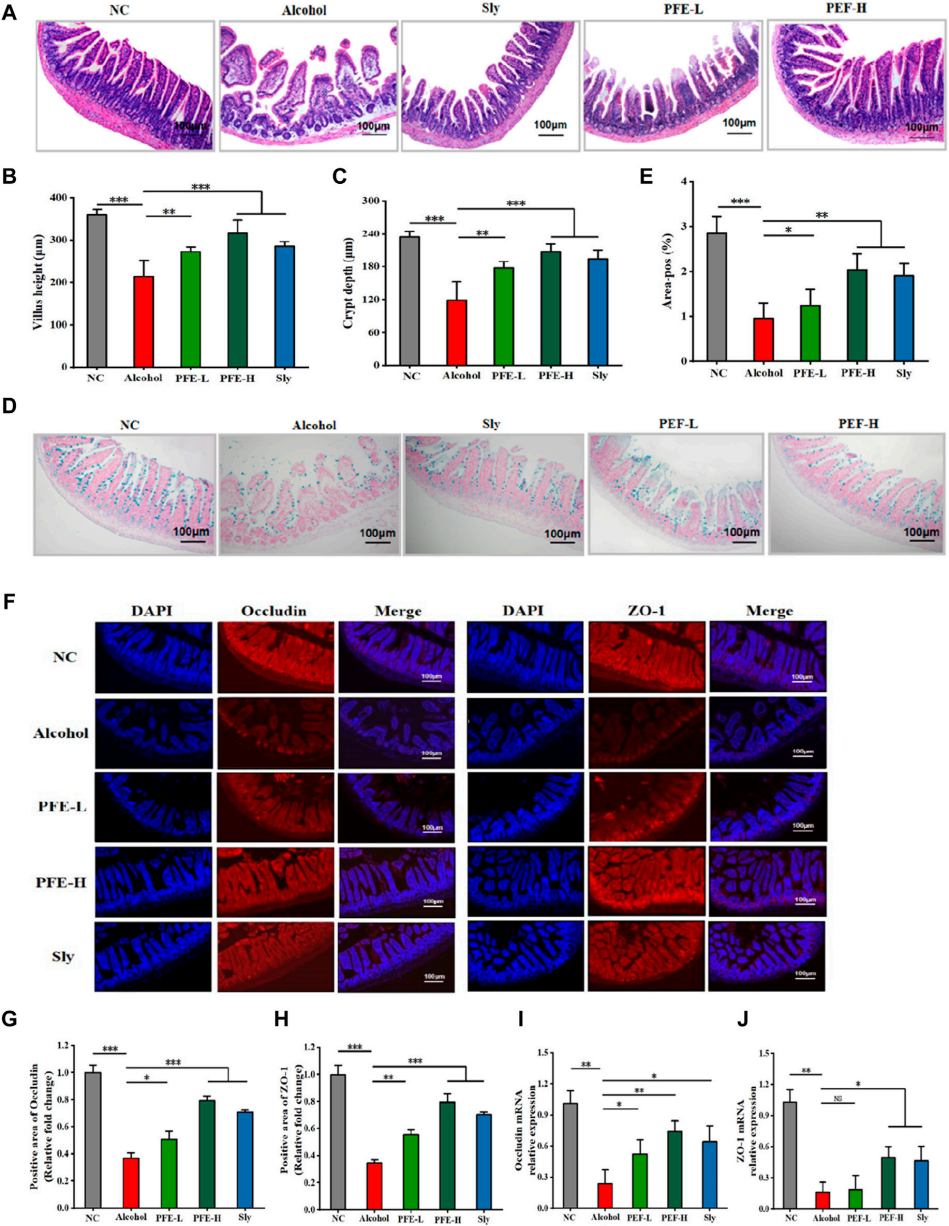
PFE preserves the integrity of the intestinal barrier in mice with alcoholic liver injury. **(A)** H&E staining of the ileum. **(B)** Quantification of ileal villus length. **(C)** Depth of the ileal crypt. **(D,E)** Representative images and quantitative analysis of Alcian blue staining for the ileum. **(F)** Representative images of ileal occludin and zonula occludens-1 (ZO-1) immunofluorescence staining of the ileum. **(G,H)** Quantitative analysis of occludin and ZO-1 proteins. **(I,J)** Relative gene expressions of occludin and ZO-1 in the ileum determined by qRT-PCR. The data are expressed as means ± SD. (*n* = 3–6). Statistical significance was determined by comparison with the alcohol group; ^*^ *p* < 0.05, ^**^ *p* < 0.01, and ^***^ *p* < 0.001. NS denotes non-significant. DAPI: 4′,6-diamidino-2-phenylindole, NC: normal control group, Alcohol: model group, Sly: silybin group, PFE-L: low-dose group, PFE-H: high-dose group.

Because LPS accumulation in the liver is closely associated with intestinal integrity (Cao et al., 2021a), we conducted a histopathological assessment and IF analysis to evaluate the ileum barrier function. Compared to the control mice, the alcohol-exposed mice exhibited significant loosening and disordering of the intestinal villi in the ileum (Figure 5A). These destructive changes were significantly attenuated after PFE intervention in the alcohol-fed mice. Furthermore, compared with the controls, the alcohol-fed mice showed notable histological changes, including detachment between crypts from the intestinal wall and reductions in both the villus height and crypt depth (Figures 5A–E), indicating that alcohol consumption altered the intestinal morphology. Fortunately, PFE administration significantly ameliorated these alcohol-induced morphological changes (Figures 5A–E). Compared to the control mice, lower expression levels of the tight junction proteins, including occludin and ZO-1 were found in the alcohol-fed mice (Figures 5F–H), which were mitigated by PFE intervention at a higher level than that achieved by positive drug treatment (Figures 5F–H). Furthermore, the qRT-PCR results showed significantly reduced mRNA expression levels of occludin and ZO-1 in response to alcohol induction when compared to those observed in the control mice (Figures 5I, J). Administration of PFE significantly upregulated the mRNA expressions of occludin and ZO-1 that were earlier diminished by alcohol exposure. The aforementioned results suggest that alcohol feeding impaired the intestinal barrier function, while PFE could effectively alleviate these injuries to maintain intestinal barrier integrity.

### 3.6 PFE alleviates alcohol-induced disturbance of the gut microbiota

**FIGURE 6:**
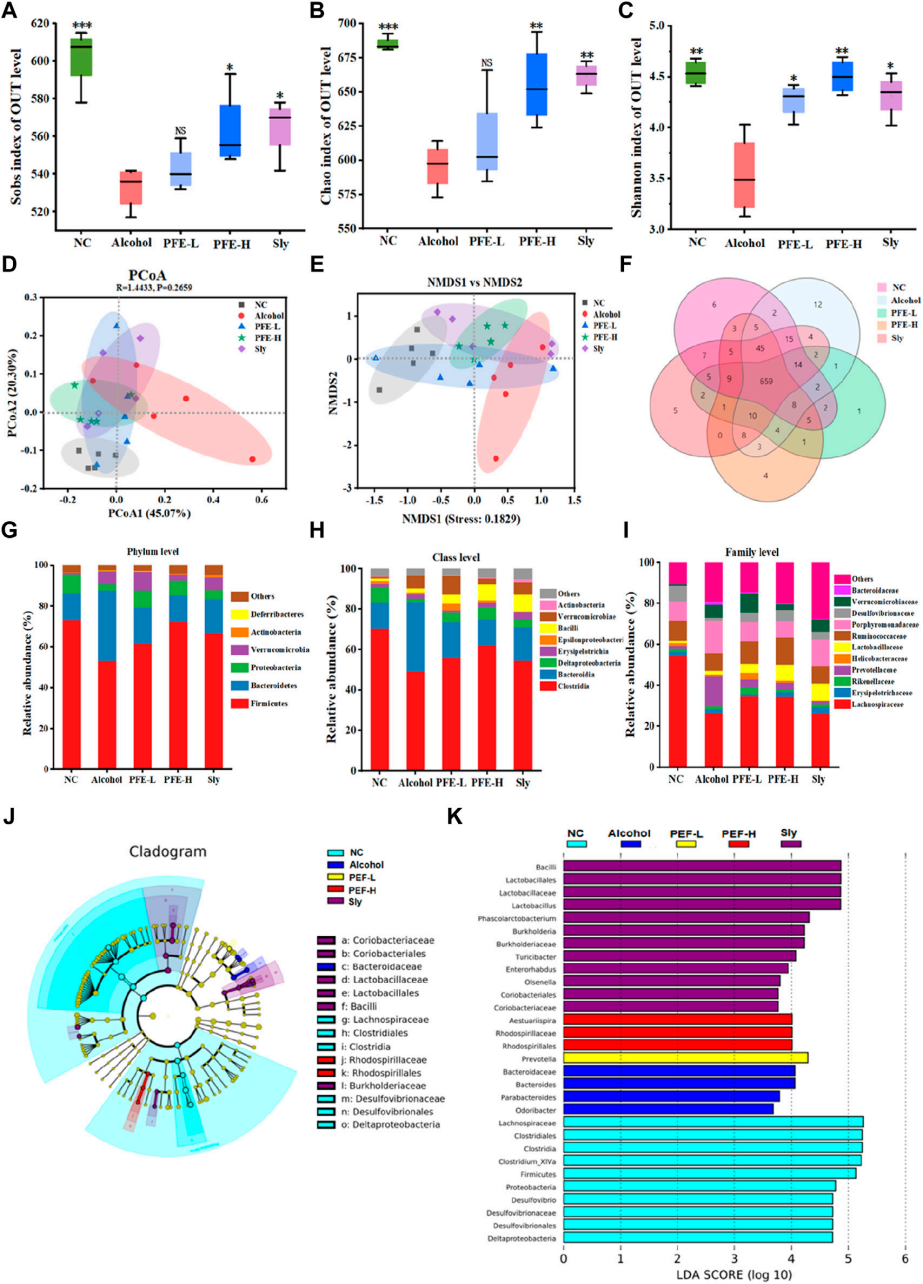
PFE alleviates alcohol-induced gut microbiota disorders in mice. **(A–C)** α-diversity assessed by Sobs index, Chao index, and Shannon index. **(D,E)** Principal coordinate analysis (PCoA) and non-metric multidimensional scaling (NMDS) analysis for β-diversity. **(F)** Venn diagram of the different groups. **(G–I)** Relative abundances of the bacteria at phylum, class, and family levels. **(J,K)** LEfSe analysis of the microbiota and its linear discriminant analysis (LDA). The data are expressed as means ± SD. (*n* = 5). Statistical significance was determined by comparison with the alcohol group; ^*^ *p* < 0.05, ^**^ *p* < 0.01, and ^***^ *p* < 0.001. NS denotes non-significant. OUT: operational taxonomic unit, NC: normal control group, Alcohol: model group, Sly: silybin group, PFE-L: low-dose group, PFE-H: high-dose group.

As intestinal dysbiosis may play a pivotal role in ALD pathogenesis, we performed 16S rDNA gene sequencing on the mouse fecal samples to analyze their microbial composition. Alpha diversity was evaluated using the Sobs, Chao, and Shannon indexes. Compared to the control mice, the alcohol-fed mice exhibited significantly decreased values of the Sobs, Chao, and Shannon indexes, indicating reduced alpha diversity induced by alcohol exposure (Figures 6A–C). PFE treatment prevented reductions of these indices induced by alcohol consumption, with better efficacy observed in the mice treated with PFE-H compared to those receiving silybin treatment. Furthermore, we employed principal coordinate analysis (PCoA) and non-metric multidimensional scaling (NMDS) analysis to identify differences in the microbial communities among the five different groups of treated mice. The results revealed that the samples from the different subjects were significantly separated, showing that the five different mice groups had significant differences in the compositions of their microbial communities (Figures 6D, E). Venn diagrams depicting the overlaps between the groups (Figure 6F) were utilized to comprehend the shared richness among these groups, with the treated groups showing similarity to the control group.

To further evaluate the effects of PFE on intestinal composition in mice with alcoholic liver injury, the bacterial compositions were analyzed at the phylum, class, and family levels. At the phylum level, *Firmicutes* and *Bacteroidetes* were dominant in each group. Compared to the control mice, the relative abundance of *Firmicutes* decreased from 73.33% to 53.24%, but the relative abundance of *Bacteroidetes* increased from 13.01% to 34.45% in the alcohol-fed mice (Figure 6G). Notably, after treatment, the relative abundances of *Firmicutes* and *Bacteroidetes* increased to 61.50% and 17.77% in mice administered PFE-L as well as 72.30% and 13.05% in mice administered PFE-H, respectively (Figure 6G). Furthermore, alcohol intake increased *Verrucomicrobia* abundance but decreased *Proteobacteria* and *Actinobacteria* relative abundances when compared to the control mice. However, PFE-H-treated mice showed significantly reduced *Verrucomicrobia* abundance as well as significantly increased relative abundances of *Proteobacteria* and *Actinobacteria* compared to the alcohol-exposed mice (Figure 6G). At the class level, compared to the controls, the relative abundances of *Bacteroidia*, *Erysipelotrichia*, *Bacilli,* and *Verrucomicrobiae* were clearly higher; however, the relative abundances of *Clostridia*, *Deltaproteobacteria*, *Epsilonproteobacteria,* and *Actinobacteria* exhibited decreases in the alcohol-treated mice. The relative abundances of these bacteria altered by alcohol exposure were restored by PFE treatment to some extent (Figure 6H). At the family level, compared to the control mice, the relative abundances of *Lachnospiraceae, Rikenellaceae, Prevotellaceae, Helicobacteraceae, Ruminococcaceae,* and *Desulfovibrionaceae* were lower, and the relative abundances of *Erysipelotrichaceae, Lactobacillaceae, Porphyromonadaceae, Verrucomicrobiaceae,* and *Bacteroidaceae* were higher in the alcohol-fed mice (Figure 6I). In turn, the relative abundances of *Lachnospiraceae, Rikenellaceae, Helicobacteraceae, Lactobacillaceae,* and *Ruminococcaceae* increased while the relative abundances of *Prevotellaceae, Porphyromonadaceae, Verrucomicrobiaceae,* and *Bacteroidaceae* decreased in the alcohol-fed mice treated with PFE when compared to mice fed with alcohol alone (Figure 6I).

To further identify the microbial and functional biomarkers from the alcohol-fed mice induced by PFE consumption, the LEfSe was performed (Figures 6J, K). The results showed that *Lactobacillus*, *Faecalibaculum,* and *Akkermansia* were the most dominant types in alcohol-fed mice treated with PFE. The administration of PFE partially restored alcohol-induced gut microbiota dysbiosis by recovering the abundance of *Lactobacillus, Faecalibaculum,* and *Akkermansia*. These findings indicate that PFE has the potential to regulate alcohol-induced dysbiosis of gut microbiota in mice.

### 3.7 PFE enriches the cecal concentrations of SCFAs in alcohol-fed mice

**FIGURE 7:**
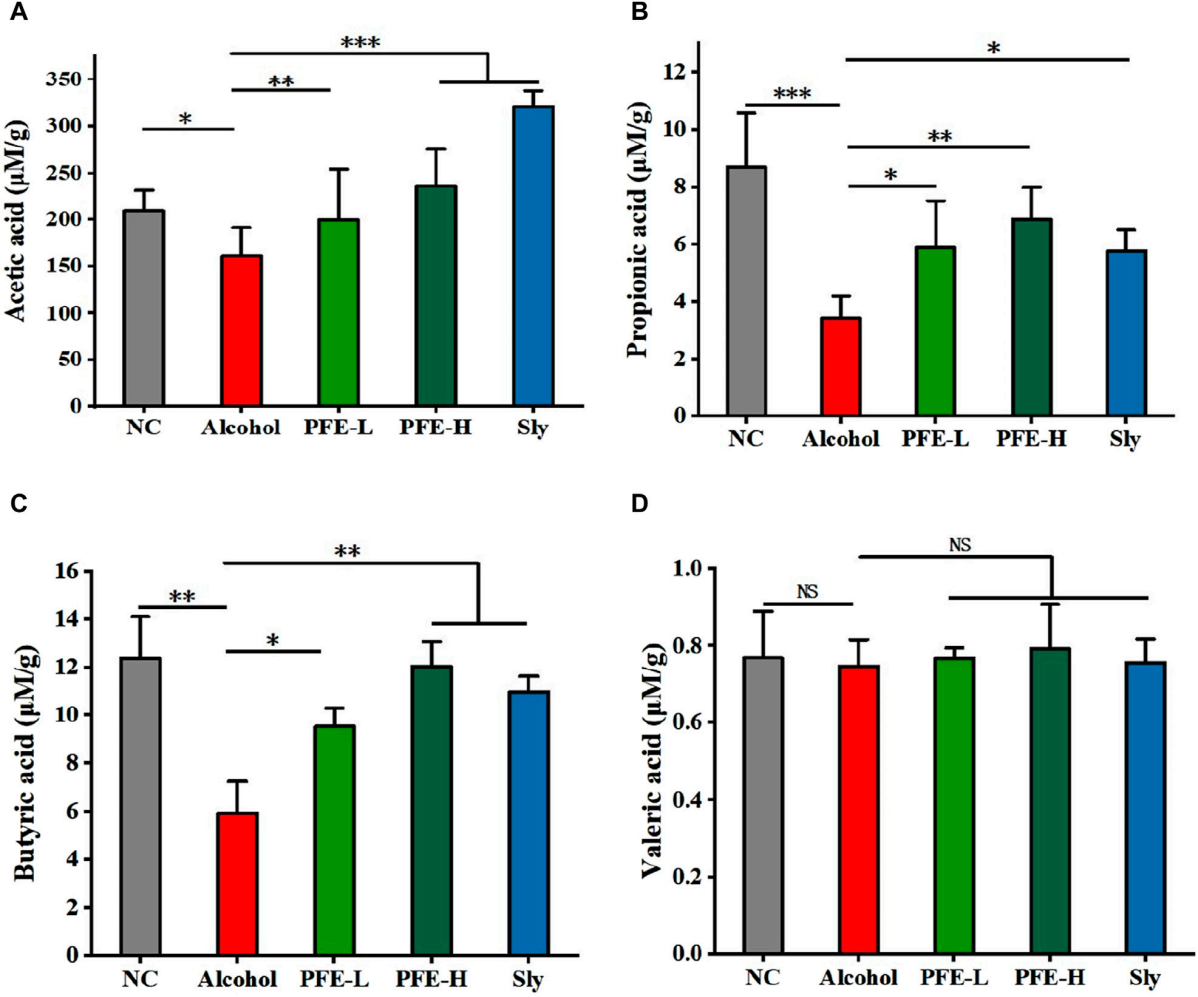
Administration of PFE facilitates production of SCFAs in mice exposed to alcohol. **(A)** Cecal levels of acetic acid. **(B)** Cecal levels of propionic acid. **(C)** Cecal levels of butyric acid. **(D)** Cecal levels of valeric acid. The data are expressed as means ± SD. (*n* = 5). Statistical significance was determined by comparison with the alcohol group; ^*^ *p* < 0.05, ^**^ *p* < 0.01, and ^***^ *p* < 0.001. NS denotes non-significant. NC: normal control group, Alcohol: model group, Sly: silybin group, PFE-L: low-dose group, PFE-H: high-dose group.

SCFAs are a group of saturated fatty acids that are produced by fermentation of non-digestible fibers, and they have been established as representative metabolites of the gut microbiota that can communicate from the microbiome to host tissues (Cait et al., 2018). In comparison to the control mice, the concentrations of acetic acid, propionic acid, and butyric acid in the ceca of the alcohol-fed mice were significantly lower (Figures 7A–C). Moreover, there was no significant difference in the valeric acid content between the control and alcohol-fed mice (Figure 7D). Notably, the concentrations of acetic acid, propionic acid, and butyric acid in the cecum were significantly higher in the alcohol-induced mice after PFE treatment (Figures 7A–C). These findings suggest that PFE exertion of an ameliorative effect on alcoholic liver injury could be related to the intestinal SCFAs.

## 4 Discussion

Excessive alcohol consumption exceeding the metabolic capacity could disrupt lipid metabolism and gut microbiota equilibrium, thereby promoting various forms of liver diseases (Wang et al., 2020b; Ren et al., 2020). Dietary interventions can effectively rectify imbalances in the intestinal microbiota, enhance the gastrointestinal physiological functions, mitigate oxidative stress, and consequently shield the liver (Gaundal et al., 2022). These interventions represent an effective strategy against ALD. PCR and FA are two well-known traditional Chinese herbal medicines having both food and medicinal functions for healthcare. However, the effects of PCR or FA treatments on alcohol-induced liver diseases and dysregulated lipid metabolism have not been reported. The present study demonstrates that an extract derived from a combination of PCR and FA (PFE) can effectively mitigate alcohol-induced liver injury and disturbance of lipid metabolism in mice. Furthermore, treatment with PFE significantly restores gut microbiota dysbiosis and maintains the integrity of the intestinal barrier, which are conducive for reducing hepatic LPS and increasing circulating SCFAs in mice exposed to alcohol. Administration of PFE was also found to sensibly suppress the TLR4/NF-κB signaling pathway while reducing hepatic inflammation induced by alcohol in mice. Our findings specifically identified that PFE could be used as a new dietary candidate for treating ALD.

ALD is characterized by liver inflammation, oxidative stress, hepatocyte metabolism dysfunction, bacterial product translocation from the gut microbiota into circulation, and regeneration process modifications (Qi et al., 2022). Numerous studies have shown that alcohol feeding induces liver injury with increased ALT and AST levels in both animals and humans (Du et al., 2022; Shen et al., 2024). Consistent with these studies, clear increases in the serum ALT and AST were observed in mice fed with alcohol. The serum levels of ALT and AST were significantly reduced after PFE treatment in the ALD mice in a dose-dependent manner, suggesting the hepatoprotective effect produced by PFE. The metabolism of ethanol directly triggers production of ROS and reactive nitrogen species, which create an environment favorable to oxidative stress (Wang and Mu, 2021b; Contreras-Zentella et al., 2022). Moreover, chronic alcohol consumption induces liver oxidative stress accompanied by depletion of GSH levels and decrease of antioxidant activity (Ge et al., 2023). In line with these findings, mice exposed to alcohol exhibited lower levels of hepatic GSH, GSH-Px, and SOD as well as higher hepatic MDA levels than the control mice. Fortunately, PFE administration significantly enhanced the hepatic levels of GSH, GSH-Px, and SOD, while also reducing hepatic MDA levels in the alcohol-fed mice, indicating that PFE has good antioxidant capacity. There is growing evidence that oxidative stress can activate fatty acid synthesis to trigger lipid droplet accumulation in the hepatocytes (Sun et al., 2020). Hepatocytes exposed to alcohol increase the NADH/NAD^+^ ratio, which can enhance lipid synthesis and prevent β-oxidation of free fatty acids, leading to triglyceride accumulation in the liver (German et al., 2021; Kasper et al., 2023). Disturbance of the lipid metabolism will result in progression of hepatic steatosis, which is the early stage of alcoholic liver injury (Jeon and Carr, 2020). The present study found significant accumulation of lipids in both the blood and liver in mice fed with alcohol. Notably, PFE administration significantly improved lipid accumulation in the alcohol-fed mice, as evidenced by the reductions in the TG and TC levels in both the liver and serum. These findings suggest that supplementation with PFE effectively prevents lipid droplet accumulation in the liver in ALD mice.

Lipid accumulation in the macrophages and other immune cells can promote inflammatory responses by augmenting TLR signaling and inflammasome activation (Tall and Yvan-Charvet, 2015; Hu, et al., 2021). NLRP3 inflammasomes can be activated by the TLR4-NF-κB axis to promote the maturation and release of downstream inflammatory cytokines while aggravating the progression of ALD (Seoane et al., 2020). In the present study, we found that the levels of LPS and inflammatory cytokines TNF-α, IL-6, and IL-1β were higher in the alcohol-exposed mice than the control mice, suggesting that alcohol-induced inflammatory responses may be triggered by excessive LPS. Exposure to LPS can induce tissue damage and organ failure (Dayang et al., 2021). Fortunately, treatment with PFE significantly reduces the levels of these inflammatory cytokines in alcohol-fed mice. Moreover, increased levels of bacterial LPS have been found to trigger a TLR4-mediated proinflammatory cascade in the immune cells (monocytes and macrophages), resulting in the activation of the NF-κB signaling pathway and leading to inflammation (Manco et al., 2010; Sun et al., 2021). Notably, in line with the changes in these inflammatory cytokines and LPS, PFE treatment also significantly inhibited activation of the hepatic TLR4/NF-κB/NLRP3 signaling pathway in mice fed with alcohol. TLR4 and NF-κB are widely recognized for their crucial roles in innate immunity and orchestration of inflammatory responses (Shao et al., 2020; Gudowska-Sawczuk and Mroczko, 2022; Zhou et al., 2023). Dysregulation of TLR4/NF-κB/NLRP3 signals have been implicated in various diseases that are primarily associated with dysfunction of the defense system and inflammation (He et al., 2022; Pu et al., 2023). It is known that activated macrophages are usually divided into M1-like and M2-like macrophages, which are individually involved in proinflammatory and anti-inflammatory responses. LPS can drive macrophage polarization to the M1 phenotype and are involved in the proinflammatory responses (Chen et al., 2020). Interestingly, PFE administration significantly regulates macrophage polarization, as indicated by the increased hepatic mRNA expression of IL-10 and decreased hepatic mRNA expressions of IL-18, iNOS, and arginase-1 in the alcohol-fed mice. This finding is supported by previous findings that the control of macrophage activation and polarization is conducive to regulating chronic inflammation (Pei et al., 2020; Zhou et al., 2022; Wu et al., 2023). Together, these findings suggest that PFE alleviates alcohol-induced hepatic inflammation possibly through inhibition of the LPS-TLR4-NF-κB/NLRP3 axis as well as modulation of macrophage activation and polarization.

Numerous studies have demonstrated that LPS are mainly produced by the gut microbiota and translocated to the portal blood stream. The composition and functions of the gut microbiota could be altered by alcohol exposure (Jung et al., 2022; Jew and Hsu, 2023). In the present study, there were significant decreases in the relative abundances of *Firmicutes*, *Proteobacteria,* and *Actinobacteria* at the phylum level in the alcohol-fed mice compared with the control mice. This finding is consistent with a previous report on the increase in relative abundance of *Proteobacteria* following alcohol consumption (Zogona et al., 2023). These bacterial changes were well reversed by PFE treatment. It has been reported that *Lachnospiraceae, Lactobacillaceae,* and *Akkermansiaceae* (the major family of *Verrucomicobiota*) can produce SCFAs. The significant absence of butyric-acid-producing bacteria and butyric acid genes in the gut causes alcohol-induced intestinal microecological dysbiosis, which plays a crucial pathogenic role in the development of alcoholic liver injury (Singhal et al., 2021). As expected, PFE treatment significantly enriches the content of SCFAs in mice exposed to alcohol. SCFAs have been considered to play important roles in modulating the integrity of the epithelial barrier by coordinated regulation of the tight junction proteins (Morrison and Preston, 2016). These proteins modulate the intracellular molecular highway between the lumen and hepatic portal system. The intestinal barrier integrity is strongly correlated with the accumulation of endotoxin LPS in the body. Increased permeability is related to translocation of the harmful bacteria and their metabolites that trigger an inflammatory cascade (Cao, et al., 2021b). In the present study, we observed that alcohol-fed mice exhibited impaired integrity of the ileum, as indicated by reduced intestinal villi and crypts than the control mice. Furthermore, the expressions of occludin and ZO-1 proteins in the ileum in the ALD mice were significantly lower than those in the control mice, suggesting a dysfunction of the intestinal barrier induced by alcohol exposure in these mice. This may also be attributed to alcohol and its major oxidative metabolite acetaldehyde, which can disrupt epithelial tight junctions (Hyland et al., 2022). Fortunately, PFE administration improved alcohol-induced impairment of the intestinal functions in mice by increasing the levels of tight junction proteins ZO-1 and occludin, which are crucial components for maintaining selective permeability against bacterial invasion and antigens. These findings indicate that PFE exerts substantial protective effects on both ALD and intestinal barrier integrity, which might at least be partly related to regulation of the gut microbiota and circulating SCFAs.

## 5 Conclusion

**FIGURE 8:**
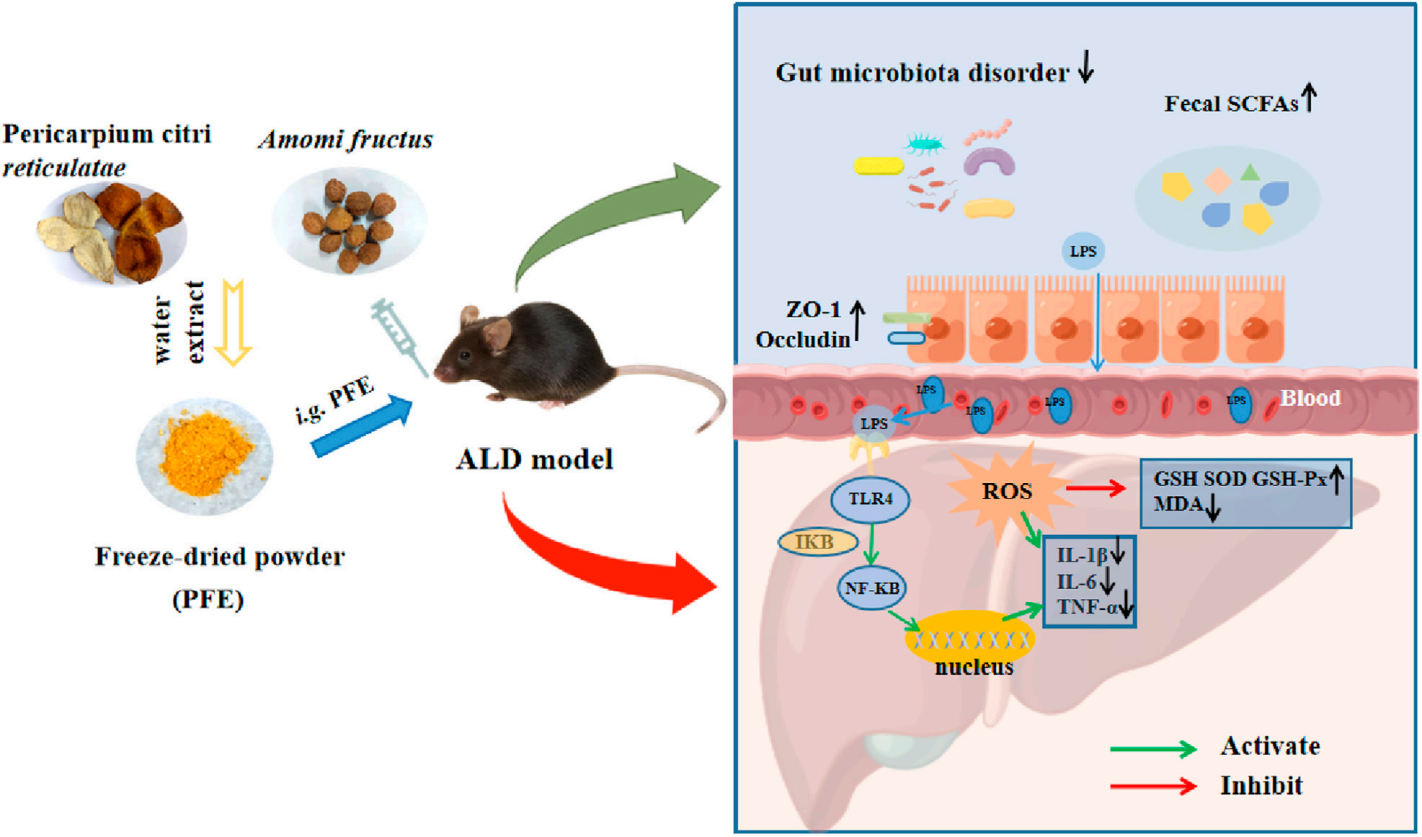
Possible protective mechanism of PFE against alcohol-induced liver injury.

PFE administration exerts strong hepatoprotective effects against metabolic disorders, oxidative damage, and inflammation induced by alcohol exposure. The amelioration of alcohol-induced hepatic inflammation by PFE treatment could be related to the decrease in bacteria-derived LPS and inactivation of the TLR4/NF-κB signaling pathway. Notably, supplementation with PFE effectively reshaped the gut microbiota and strengthened the intestinal barrier in ALD mice, which helped prevent the translocation of LPS into the blood and liver (Figure 8). These findings offer novel insights into the underlying mechanisms by which PFE prevents ALD. PFE can thus serve as an effective clinical candidate for minimizing the risks of various ALD. However, the detailed mechanisms by which PFE improves ALD will need more work in the future. For instance, fecal microbial transplantation may be used to identify the roles of both gut microbiota and specific strains in the anti-ALD of PFE. Furthermore, the anti-ALD of specific strains alone or in combination with PFE can also be explored using germ-free animals.

## Data Availability

All data included in this study are available upon request by contacting the corresponding authors. The 16S rRNA data generated in this study were deposited in the NCBI (SRA) database; accession number PRJNA1123434, available at: http://www.ncbi.nlm.nih.gov/bioproject/1123434.
